# Microsatellite analysis of Damask rose (*Rosa damascena *Mill.) accessions from various regions in Iran reveals multiple genotypes

**DOI:** 10.1186/1471-2229-7-12

**Published:** 2007-03-08

**Authors:** Alireza Babaei, Seyed Reza Tabaei-Aghdaei, Morteza Khosh-Khui, Reza Omidbaigi, Mohammad Reza Naghavi, Gerhard D Esselink, Marinus JM Smulders

**Affiliations:** 1 Department of Horticultural Science, Faculty of Agriculture, Tarbiat Modares University, P.O. Box 14115-365, Tehran, Iran; 2 Biotechnology Research Department of Natural Resources, Research Institute of Forests and Rangelands, P.O. Box 13185-116, Tehran, Iran; 3 Department of Horticultural Science, Faculty of Agriculture, Shiraz University, Shiraz, Iran; 4 Department of Plant Breeding, Faculty of Agriculture, University of Tehran, Tehran, Iran; 5 Plant Research International, Wageningen UR, P.O. Box 16, 6700 AA Wageningen, The Netherlands

## Abstract

**Background:**

Damask roses (*Rosa damascena *Mill.) are mainly used for essential oil production. Previous studies have indicated that all production material in Bulgaria and Turkey consists of only one genotype. Nine polymorphic microsatellite markers were used to analyze the genetic diversity of 40 accessions of *R. damascena *collected across major and minor rose oil production areas in Iran.

**Results:**

All microsatellite markers showed a high level of polymorphism (5–15 alleles per microsatellite marker, with an average of 9.11 alleles per locus). Cluster analysis of genetic similarities revealed that these microsatellites identified a total of nine different genotypes. The genotype from Isfahan province, which is the major production area, was by far the most common genotype (27/40 accessions). It was identical to the Bulgarian genotype. Other genotypes (each represented by 1–4 accessions) were collected from minor production areas in several provinces, notably in the mountainous Northwest of Iran.

**Conclusion:**

This is the first study that uncovered genetic diversity within Damask rose. Our results will guide new collection activities to establish larger collections and manage the Iranian Damask rose genetic resources. The genotypes identified here may be directly useful for breeding.

## Background

There are almost 200 species and more than 18000 cultivars in the genus *Rosa *[1]. They are mostly shrubs, distributed in the temperate zones of the Northern hemisphere [2]. One of the important *Rosa *species is *Rosa damascena *Mill., which is commercially used for essential oil production and cultivated as garden rose [3]. In recent years, antioxidant, antibacterial and antimicrobial activities of *R. damascena *essential oil have been demonstrated [4-7]. Three recent studies on molecular analyses of genetic diversity of *Rosa damascena *Mill. with RAPD, AFLP and SSR markers did not show any polymorphism among *R. damascena *plants from various plantations in Turkey[8,9] and Bulgaria[3], indicating that commercial production of essential oil is in fact done by large scale propagation of only one or very few genotypes.

*R. damascena *can now be found in the wild in Morocco, Andalusia, the Middle East, and the Caucasus. As Damask roses were originally introduced from the Middle East into Western Europe, it is thought that the origin and centre of diversity of Damask roses can be found in this region. In Iran, cultivation and consumption of Damask roses has a long history. Crude distillation of roses was probably developed in Persia in the late 7^th ^century A.D. [3,10-12].

In order to study genetic diversity of *R. damascena *in Iran, all relevant geographical regions of Iran were sampled. Some samples were taken from large production fields in the main rose oil production area in the centre of the country, but most of the samples were collected from smaller production fields and abandoned fields in remote and mountainous areas. In this way 40 Damask rose accessions were collected from 28 provinces of Iran. Results on morphology and oil content variation suggest that this collection may include multiple genotypes [13].

In this investigation, a microsatellite marker analysis of the Iranian collection of *R. damascena *is reported. We show that we have obtained as much as nine different genotypes, of which some have been used for regional production of Damask rose oil.

## Results

### Microsatellite analysis

In this study 40 accessions of *Rosa damascena *(Table 1) that showed a high level of phenotypic and oil content variation were analyzed with nine microsatellite markers. All markers detected polymorphisms among the samples. The number of alleles ranged from 5 to 15 with an average of 9.11 (Table 2). Using the MAC-PR method, we determined the allelic configurations at six loci (RhP519, RhB303, RhEO506, RhD221, RhP50, RhE2b) for all investigated accessions (Table 3).

### Genotype identification

**Figure 1:**
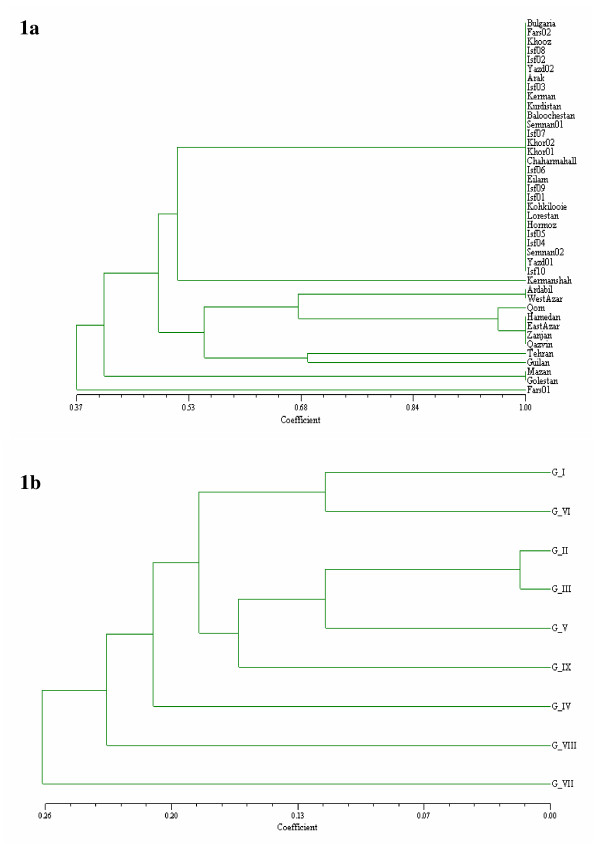
1a UPGMA clustering of Dice genetic similarities based on dominant scores of microsatellite alleles, among all accessions of Damask rose included in this study. Note that 1 (similarity) = genetically identical. 1b UPGMA clustering of genetic distances based on pairwise Fst among the nine unique genotypes, derived from codominant scores of six microsatellite loci. Note that 0 (distance) = genetically identical.

Cluster analysis resulted in grouping of the 40 accessions into nine distinct genotypes (Fig. 1). The main group consisted of 27 landraces that showed the same microsatellite profile. This group included all accessions from the main rose oil production sites of Damask rose in Iran. The pattern of this group was identical to that of an accession from Bulgarian production areas. Rusanov et al. showed that all Bulgarian Damask roses are this genotype [3].

The other genotypes that we identified in the cluster analyses were present in much smaller numbers. Some genotypes were unique (accessions from Tehran, Guilan, Kermanshah, Qom provinces and one accession from Fars province); others were present as two or four accessions (Fig. 1a and Table 1). The unique accessions were from mountainous and remote areas in the Northwest of Iran where roses are cultivated on small scale. In addition, the accessions from the humid area near the Caspian Sea were different from all other accessions as well.

The accessions from Fars province formed two distinct clusters in the dendrogram. They are from an environmentally very distinct region, far to the South of Iran. One of these samples was hexaploid, while all other samples were tetraploid, as expected for *R. damascena*.

As expected, the absolute magnitude of genetic distances based on codominant scoring is much smaller than that of dominant scores, as more alleles are shared, but the topologies of the trees (Figure 1a and Figure 1b) are largely comparable for those samples that were not too genetically distant.

## Discussion

It seems that for commercial rose production only one and the same genotype is used in several countries. This makes it likely that also in Turkey this genotype is being used for large-scale production, but this remains to be confirmed as samples from Turkey were not included in the study of Rusanov et al. [3] nor in the present study.

Except one plant, all genotypes identified here were tetraploid, consistent with the general literature. One plant was hexaploid. At this moment, we do not know whether this is the first of more hexaploid *R. damascena *plants. It may be misclassified, but cuttings from all plants have been evaluated by several experienced taxonomists after cultivation for 2–3 years in a common garden.

The genetic distances among accessions were not correlated with geographical distances among their places of origins (not shown). Clearly, a larger sample of genotypes will be necessary to determine whether there is some relationship with geographical distance, whether there is isolation of populations due to barriers in gene flow, or whether different climatic conditions lead to differentiation within the species.

In MAC-PR analysis we determined the allelic configuration based on six loci, because in the other three loci, not all alleles were present in plants in completely heterozygous configurations, which is necessary to be able to accurately determine the relative amplification of each allele [16]. Genotype G_II and G_III differ by only one allele at locus RhEO506. This is surprising as genotypes in roses are usually identical (due to vegetative propagation) or very different (due to segregation of alleles from the heterozygous parents) [17]. Remarkably, this small difference is confirmed in the MAC-PR analysis, as no differences were found in allele frequencies at the other five loci. Although this does not completely rule out that the two plants are close relatives, a mutation leading to an allele that is one repeat longer is a more likely possibility. Genotype G_III was from Qom, which borders the three provinces in which genotype G_II was found.

## Conclusion

Our analysis showed for the first time the existence of multiple genotypes within *Rosa damascena*. We are currently performing an analysis of oil production across several years, in order to determine whether different genotypes also have a qualitative difference in production and/or composition of essential oil. If so, these genotypes may be used to broaden the production of rose oil, and they can also be used as the basis of a breeding program. As these nine genotypes were found after sampling only 40 large and small production fields, we expect that a more intensive sampling will be valuable in order to find more genetic diversity. For this, we will focus on the areas where we have found the unique genotypes, i.e., the Western and Northern provinces.

## Methods

### Plant material

A total of 40 Damask rose accessions were collected from 28 provinces of Iran (Table 1), in order to obtain a good geographical coverage of the country and a good coverage of the 13 different climatic regions that have been identified [13]. Samples were taken from commercial production fields and from small (< 5 ha) or abandoned production fields. All accessions were grown from 2000 onwards in experimental field of the Research Institute of Forests and Rangelands (RIFR), Tehran, Iran. DNA was extracted from fresh young leaves using the Qiagen DNeasy Plant Mini Kit (Westburg, The Netherlands).

### Microsatellite analysis

A set of nine robust microsatellite markers were selected from Esselink et al. [17] and Yan et al. [15] representing different linkage groups on the genetic map of rose (Table 2). These markers are highly polymorphic in hybrid tea rose [17] and in other *Rosa *species [18-20], and hence have a high discriminative power to differentiate genotypes. Fluorescently labelled (6FAM, HEX or NED) primer pairs were amplified in three multiplexes using the Qiagen PCR multiplex kit (Westburg, The Netherlands). The PCR program for amplification were as follows: 94°C for 15 min; 30 cycles of 94°C for 30 s, ramp to 50°C (1°C/s), 50°C for 30 s, ramp to 72°C (1°C/s), 72°C for 2 min; and a final elongation step at 72°C for 10 min. Fluorescent amplification products were detected using an ABI Prism 3700 DNA Analyzer (Applied Biosystems) and all samples were genotyped in accordance with reference alleles for each locus as described by Vosman et al. [21], using Genotyper 3.5 NT (Applied Biosystems).

### MAC-PR and statistical analysis

The microsatellite DNA allele counting – peak ratios method (MAC-PR), which was developed for the tetraploid hybrid tea rose (*Rosa × hybrida *L.) varieties by Esselink et al. [16], assigns precise allelic configurations (the actual genotype) based on quantitative values for peak areas provided by the Genotyper software. For each locus, all alleles were analyzed in pairwise combinations in order to determine their copy number in the individual samples. This was accomplished by calculating ratios between the peak areas for two alleles in all samples in which these two alleles occurred together.

Genetic distances were calculated either as Dice similarities on the basis of dominant scoring of individual alleles in NTSYS 2.1 (Applied Biostatistics) or as pairwise Fst of the MAC-PR genotypes using SPAGeDi 1.2 [22]. The use of Dice (Nei & Li) coefficient is more suitable for codominant markers such as SSRs when they are scored dominantly [23,24]. The accessions were clustered using the unweighted pair group method using arithmetic averages (UPGMA) module of NTSYS.

## Authors' contributions

SRTA established the Damask rose collection. AB, SRTA, MKK, MRN and RO designed the study. AB selected plant material and performed DNA extraction. AB, GDE and MJMS performed SSR and data analysis. AB, MJMS, GDE and MRN wrote the primary draft. All authors were involved in the final version of the paper.

## Figures and Tables

**Table 1:** Geographical origins of Iranian Damask rose accessions

Origin site no.	Province(s) included	Accession name	Climate^a^	Genotype^b^
Os1	Isfahan	Isf01	Cool temperate – semi arid	G_I
		Isf02		G_I
		Isf03		G_I
		Isf04		G_I
		Isf05		G_I
		Isf06		G_I
		Isf07		G_I
		Isf08		G_I
		Isf09		G_I
		Isf10		G_I
Os2	East & West Azarbayjan, Ardabil	EastAzar	Cool temperate – semi arid	G_II
		WestAzar		G_V
		Ardabil		G_V
Os3	Kermanshah, Eilam	Kermanshah	Temperate – semi humid	G_VII
		Eilam		G_I
Os4	Tehran, Markazi	Tehran	Cool temperate – semi arid	G_VI
		Arak		G_I
Os5	Chaharmahall, Kohkilooie, Lorestan	Chaharmahall	Temperate – semi arid	G_I
		Kohkilooie		G_I
		Lorestan		G_I
Os6	Razavi Khorasan, South Khorasan	Khor01	Temperate – arid	G_I
		Khor02		G_I
Os7	Khoozestan, Hormozgan, Baloochestan	Khooz	Warm – arid	G_I
		Hormoz		G_I
		Baloochestan		G_I
Os8	Zanjan, Qazvin	Zanjan	Cool temperate – semi arid	G_II
		Qazvin		G_II
Os9	Semnan, Qom	Semnan01	Warm temperate – arid	G_I
		Semnan02		G_I
		Qom		G_III
Os10	Fars, Kerman	Fars01	Temperate – semi arid	G_IX
		Fars02		G_I
		Kerman		G_I
Os11	Kurdistan, Hamedan	Kurdistan	Cool – semi arid	G_I
		Hamedan		G_II
Os12	Guilan, Mazandaran, Golestan	Guilan	Temperate – humid	G_VIII
		Mazan		G_IV
		Golestan		G_IV
Os13	Yazd	Yazd01	Warm temperate – arid	G_I
		Yazd02		G_I

^a ^Yearly mean temperature in warm, temperate and cool climates are 15–25°C, 10–15°C and 0–5°C, respectively. Yearly mean rainfalls in semi humid, semi arid and arid climates are 600–1400 mm, 300–600 mm and 100–300 mm, respectively.

^b^Genotypes as identified in this study

**Table 2:** Characteristics of the microsatellite markers used.

Locus	Label	Linkage group^a^	Number of alleles
RhP519	6FAM	n.d.^b^	6
RhB303	HEX	n.d.^b^	11
RhO517	NED	1	5
RhEO506	6FAM	2	13
RhD221	HEX	4	7
RhAB73	NED	7	9
RhP50	6FAM	3	15
RhAB40	HEX	4	8
RhE2b	NED	6	8
Average			9.11

^a ^from Debener et al. [14] and Yan et al. [15]

^b ^n.d.= not determined

**Table 3:** Allele configuration of the nine different *R. damascena *genotypes based on MAC-PR analyses

**Genotype**	**Number of accessions**	**Marker**					
		RhP519	RhB303	RhEO506	RhD221	RhP50	RhE2b

G_I	27	232 232 232 232	119 125 127 128	210 222 228 260	209 217 223 226	349 371 404 420	168 170 177 180
G_II	4	219 232 232 241	127 128 128 130	207 213 219 260	209 217 217 223	326 363 371 396	168 168 177 182
G_III	1	219 232 232 241	127 128 128 130	207 213 219 263	209 217 217 223	326 363 371 396	168 168 177 182
G_IV	2	219 232 232 238	122 127 127 129	213 213 228 240	200 209 220 223	343 371 433 433	168 180 180 189
G_V	2	232 232 232 241	127 128 128 130	210 213 213 228	211 217 217 223	349 363 371 396	168 168 180 189
G_VI	1	219 232 232 232	119 125 127 127	210 213 222 228	209 211 223 223	343 371 396 404	168 177 180 189
G_VII	1	219 219 232 238	122 125 145 146	222 225 228 228	217 223 226 226	374 374 404 404	168 168 174 189
G_VIII	1	219 219 232 232	119 125 127 129	213 213 228 228	211 211 223 223	326 354 396 396	180 180 189 189
G_IX	1	219 219 232 232 247 235	117 119 125 128 129 133	195 201 213 234 246 260	211 217 220 220 223 232	340 354 380 396 399 411	168 168 180 182 189 199
